# Visual stimulation and frequency of focal neurological symptoms engage distinctive neurocognitive resources in migraine with aura patients: a study of resting-state functional networks

**DOI:** 10.1186/s10194-022-01446-4

**Published:** 2022-07-12

**Authors:** Gianluca Coppola, Ilenia Corbelli, Antonio Di Renzo, Andrea Chiappiniello, Pietro Chiarini, Vincenzo Parisi, Giorgio Guercini, Paolo Calabresi, Roberto Tarducci, Paola Sarchielli

**Affiliations:** 1 Department of Medico-Surgical Sciences and Biotechnologies, Sapienza University of Rome Polo Pontino – I.C.O.T., Via Franco Faggiana 1668, 04100 Latina, Italy; 2 Section of Neurology, Department of Medicine and Surgery, University of Perugia, Perugia, Italy; 3 IRCCS – Fondazione Bietti, Rome, Italy; 4 Medical Physics Service, Azienda Ospedaliera Di Perugia, Perugia, Italy; 5 Neuroradiology Service, Azienda Ospedaliera Di Perugia, Perugia, Italy; 6 Department of Neuroscience, Cattolica Sacro Cuore University, Rome, Italy; 7 Neurologia, Fondazione Policlinico Universitario A. Gemelli IRCCS, Rome, Italy

**Keywords:** Aura, Attentive systems, Executive control network, Salience network, Decision-making

## Abstract

**Introduction:**

Several functional neuroimaging studies on healthy controls and patients with migraine with aura have shown that the activation of functional networks during visual stimulation is not restricted to the striate system, but also includes several extrastriate networks.

**Methods:**

Before and after 4 min of visual stimulation with a checkerboard pattern, we collected functional MRI in 21 migraine with aura (MwA) patients and 18 healthy subjects (HS). For each recording session, we identified independent resting-state networks in each group and correlated network connection strength changes with clinical disease features.

**Results:**

Before visual stimulation, we found reduced connectivity between the default mode network and the left dorsal attention system (DAS) in MwA patients compared to HS. In HS, visual stimulation increases functional connectivity between the independent components of the bilateral DAS and the executive control network (ECN). In MwA, visual stimulation significantly improved functional connectivity between the independent component pairs salience network and DAS, and between DAS and ECN. The ECN Z-scores after visual stimulation were negatively related to the monthly frequency of aura.

**Conclusions:**

In individuals with MwA, 4 min of visual stimulation had stronger cognitive impact than in healthy people. A higher frequency of aura may lead to a diminished ability to obtain cognitive resources to cope with transitory but important events like aura-related focal neurological symptoms.

## Introduction

Migraine is characterized by abnormal processing of visual cues both during and between attacks [1, 2]. This is more evident in migraine patients with aura (MwA), who are functionally more hyperresponsive to visual stimulation [3], with greater activation of the visual BOLD signal and associated with greater discomfort to light [4, 5], implying a link between vision and cognitive emotion processing. This link has been revealed in healthy individuals using both task-related and resting state (rs) functional magnetic resonance imaging (fMRI) studies, which revealed that visual stimulation activates not only the primary visual area, but also other distant brain areas likely to be involved in the emotional and cognitive-behavioral components [6–9]. The precentral gyri, middle frontal gyri, and superior and inferior temporal gyri are the most noteworthy extrastriate regions that were discovered to be functionally related following visual stimulation [8]. All of these regions are part of multiple neurocognitive networks that have previously been linked to MwA pathogenesis [10–17], and their dysfunction may explain why MwA patients appear to have more pronounced cognitive abnormalities than migraine without aura [18]. According to this research, neurocognitive networks may exhibit altered functional connectivity in response to visual stimulation in the context of a state of impaired visual information processing, such as in MwA patients.

In this study, we used rs-fMRI and independent component analysis (ICA) to investigate the potential role of visual stimulation in modulating the interictal connectivity of cortical networks in patients with MwA by recording rs-fMRI before and after 4 min of visual stimulation with a checkerboard pattern. We postulate that, in MwA, the visual pattern may produce distinct functional connectivity across large-scale interacting neural networks, and that this may be affected by the recurrence of aura symptoms.

## Patients and methods

This non-pharmacological interventional study was approved by our Institutional Review Board and local Ethical Committee [M-Image 2609/15]. An informed consent was obtained from all participants. The study complied with the principles set out in the WMA Declaration of Helsinki- Ethical principles for medical research involving human subjects.

### Participants

All patients were enrolled at the Headache Centre of the Neurologic Clinic, University of Perugia, Italy. Inclusion criteria for patients were as follows: (i) patient diagnosed with episodic (no more than 6 days/month with headache) migraine with aura (MwA) according to ICHD-III criteria [2]; (ii) aged 18–65 years. Healthy subjects (HS), of comparable age and sex, in non-menstrual period (if female), were recruited among patients’ friends and hospital’s staff. They were not recruited if they had a history of migraine (she/he and their first-degree relatives) and/or history of migraine equivalents or other primary headaches. Exclusion criteria, common for all participants, were as follows: (i) absolute contraindications to MRI; (ii) other neurological disease; (iii) acute or chronic pain in progress; (iv) history of febrile convulsions and/or epilepsy or epileptic first-degree relatives. Moreover, we excluded participants with neuro-ophthalmological disorders by testing them on a complete neuro-ophthalmological examination, which included assessment of visual acuity, intraocular pressure measurement, split lamp biomicroscopy, and indirect ophthalmoscopy. All patients were pain free for at least 3 days before the recording session and for at least 3 days after. All patients had not been taking prophylactic medications for at least 3 months. Patients and HS were recruited from October 2015 to December 2017.

For each participant, we collected data about: (i) demographics, (ii) comorbidities, (iii) migraine clinical characteristics, such as migraine onset, years of disease history, mean monthly attacks frequency, attacks duration, type, duration, days passed from the last migraine attack, and monthly frequency of migraine aura and its trigger factors, severity of headache phase (1–3 scale score). All patients were assessed by the Beck depression inventory (BDI), state-trait anxiety inventory (STAI), and Toronto alexithymia scale (TAS-20).

### fMRI protocol and visual stimuli

Patients and HS underwent MRI acquisition on a 3.0 T Philips Achieva clinical system, using an 8-channel head-coil. The acquisition protocol included a 3D sagittal T1 (FOV 240 × 240 mm^2^, 176 slices, voxel size 1 × 1x1 mm^3^, TE 3.7 ms, TR 8 ms, TI 993 ms, FA 9°, no fat suppression). Rs-fMRI data were obtained using an axial EPI gradient-echo (FOV 240 × 240 mm^2^, 40 slices, voxel size 3 × 3x3 mm^3^, 150 volumes, TE 30 ms, TR 3000 ms, SPIR fat suppression). Two rs-fMRI acquisitions were performed before and after a 4 min visual stimulus presented using E-PRIME software (Psychology Software Tools, Inc., Pittsburgh, PA, USA) and designed to display a 4 Hz flashing-checkerboards for 15 s and 15 s fixation (flickering checkerboard pattern). Each of the rs-fMRI acquisition was collected in a 7 m 30 s run, during which subjects were instructed to relax, avoid motion, and keep their eyes closed.

### fMRI data processing and analysis

Image data processing was performed using SPM12 (fil.ion.ucl.ac.uk/spm/), GIFT (trendscenter.org/software/gift/) v4.0b and FNC (trendscenter.org/trends/software/fnc/index.html) in MATLAB environment (mathworks.com/products/matlab.html).

The two rs-fMRI data-series of each subject were preprocessed with SPM 12 involving the following steps: (i) realignment using a least square approach and a 6-parameter rigid body process and reslicing by a cubic spline interpolation; (ii) co-registration with the corresponding 3D structural T1 data; (iii) spatial normalization to the Montreal Neurological Institute (MNI152) template and transformation into a common stereotactic space [19], resampled by 3 mm on each direction; (iv) spatial smoothing with an isotropic 8 mm full-width at half maximum (FWHM) Gaussian kernel.

Grouped spatial Independent Component Analysis (ICA) was performed using the infomax algorithm [20] separately on HS and patient datasets (18 HC and 21 MwA patients) including both the within-session rs-fMRI acquisitions (pre and post visual stimulus). The modified version of the minimum description length (MDL) criterion implemented by GIFT [21] was adopted to determine the number of Independent Components (ICs) (23 IC for HS and 19 IC for patients) from the two datasets. Three expert physicists-neuroradiologists (A.C., G. G., and R.T.) visually inspected all ICs blindly, plotting them to templates in GIFT using a priori probabilistic maps. They discarded ICs located in CSF or white matter, or with low correlation to gray matter that can be connected to artifacts, such as eye movements, head motion, and ballistic artifacts [22, 23].

This blind process resulted in 8 meaningful ICs for HS: default mode network (IC2), medial visual (IC7), sensory motor system (IC8), left (IC9) and right (IC10) dorsal attention stream (DAS), lateral visual (IC11), executive control (IC12) (ECN), and visuo-spatial networks (IC21). Whereas, in MwA patients the process resulted in 7 ICs: salience network (SN, IC2), sensory motor network (IC5), lateral visual network (IC6), dorsal attention system (IC10), executive control (IC12), medial visual (IC16), and default mode networks (IC17).

We performed again grouped spatial IC analysis between pre stimulation subject groups using the same GIFT’s procedure described before, resulting in 7 meaningful ICs: lateral visual (IC2), DMN (IC3), sensory motor (IC4), left DAS (IC10), medial visual (IC13), right DAS (IC14), and primary visual networks (IC16).

The above-mentioned networks were processed by means of FNC toolbox in three separate sessions respectively for pre stimulation HS and MwA groups together, HS and MwA groups.

With FNC toolbox, the resulted component time courses were band-pass filtered in the 0.033–0.13 Hz frequency range. According to the method published elsewhere [24], correlation and lag for each pair of resulted ICs were computed for patients and HCs, pre and after visual stimulation.

We extrapolated Z-scores of each IC associated spatial map, which reflects the strength of correlation of each voxel time-course with the specific IC temporal waveform [25]. To search for a correlation between rs-fMRI activation differences and patients with MwA clinical features, the voxel-wise Z-max scores of each IC network were obtained for each subject.

### Statistical analysis

Group differences for demographic data were estimated using 2-sample t-test and Chi-square test.

Two statistically significant differences in correlation between networks of pre and post visual stimulus for HS and for patients groups were identified using a 2-sample t-test with a *p* value of 0.01 and False Discovery Rate (FDR) correction, performed by FNC toolbox.

No statistically significant difference in correlation between groups’ pre-stimulation networks was found (*p* < 0.01, FDR corrected).

A reduced different (*p* < 0.05, FDR corrected) correlation was found between IC3 and IC10 in pre stimulation groups.

Moreover, connectivity combinations with statistically significant (*p* < 0.01 with FDR correction) lag values were also investigated using a paired 2-sample t-test of the difference between pre and post stimulation for HS and MwA patient lags and an unpaired 2 sample t-test between pre stimulation groups’ lags. None of the latter statistical inferences showed significant lag difference.

Correlations between demographic, clinical variables and each significant IC Z-scores maximum values were performed by mean of Pearson’s correlation test. Spearman Rho was performed for discrete variable. A significance threshold *p* < 0.05 was adopted.

## Results

We enrolled 25 MwA patient and 18 HS. Of these, 4 subjects (MwA patients) did not complete the scanning sessions, due to technical problems. All patients were right-handed and had a normal neurological examination. No demographical and psychometric differences were reported between groups (see Tables 1 and 2). Structural brain MRIs were normal in all participants. None reported adverse events from MRI scanning.

**Table 1:** Demographic and clinical features of study’s participants. Inferential statistics based on Student’s *t*-test and Chi-square method

	HS (*n* = 18)	MA (*n* = 21)	Statistics
Age	28.9 ± 7.0	29.5 ± 6.3	T = -0.71, *p* = 0.556
Female	16	17	Chi^2^ = 0.469, *p* = 0.493
BMI	21.0 ± 2.9	22.7 ± 4.6	T = -1.23, *p* = 0.226
Migraine onset (y)		15.3 ± 8.5	
Attacks frequency (n/month)		3.1 ± 2.4	
Severity of headache (1–3)		2.7 ± 0.5	
Days from the last attack (n)		9.4 ± 3.1	
Attack duration (h/month)		26.0 ± 12.1	
Aura duration (min)		36.4 ± 17.4	
Aura frequency (n/month)		1.16 ± 0.74	
Aura triggering migraine (n)		11 (52.4%)	
Visual aura		20 (95.2%)	
Sensory aura		10 (47.6%)	
Language aura		7 (33.3%)	

*HS *Healthy subjects, *MwA* Patients with migraine with aura

**Table 2:** Psychometric parameters of study’s participants. Inferential statistics based on Student’s *t*-test

	HS (*n* = 18)	MwA (*n* = 21)	Statistics
BDI tot	4.1 ± 7.4	7.5 ± 4.8	T = -1.60, *p* = 0.120
TAS-20	41.4 ± 12.4	44.4 ± 10.4	T = -0.50, *p* = 0.619
STAI T	34.7 ± 12.7	39.0 ± 9.7	T = -1.1, *p* = 0.290
STAI S	25.6 ± 4.3	28.9 ± 7.8	T = -1.1, *p* = 0.293

*HS *Healthy subjects, *MwA P*atients with migraine with aura

### Resting state functional MRI

Prior to visual stimulation, we found a significant positive correlation of the independent component IC3 and IC10, corresponding to the DMN and left DAS respectively, in both the HS group (*r* = 0.33, *p* < 0.001) and the MwA group (*r* = 0.16, *p* < 0.001) at *p* < 0.01 FDR corrected.

**Fig. 1:**
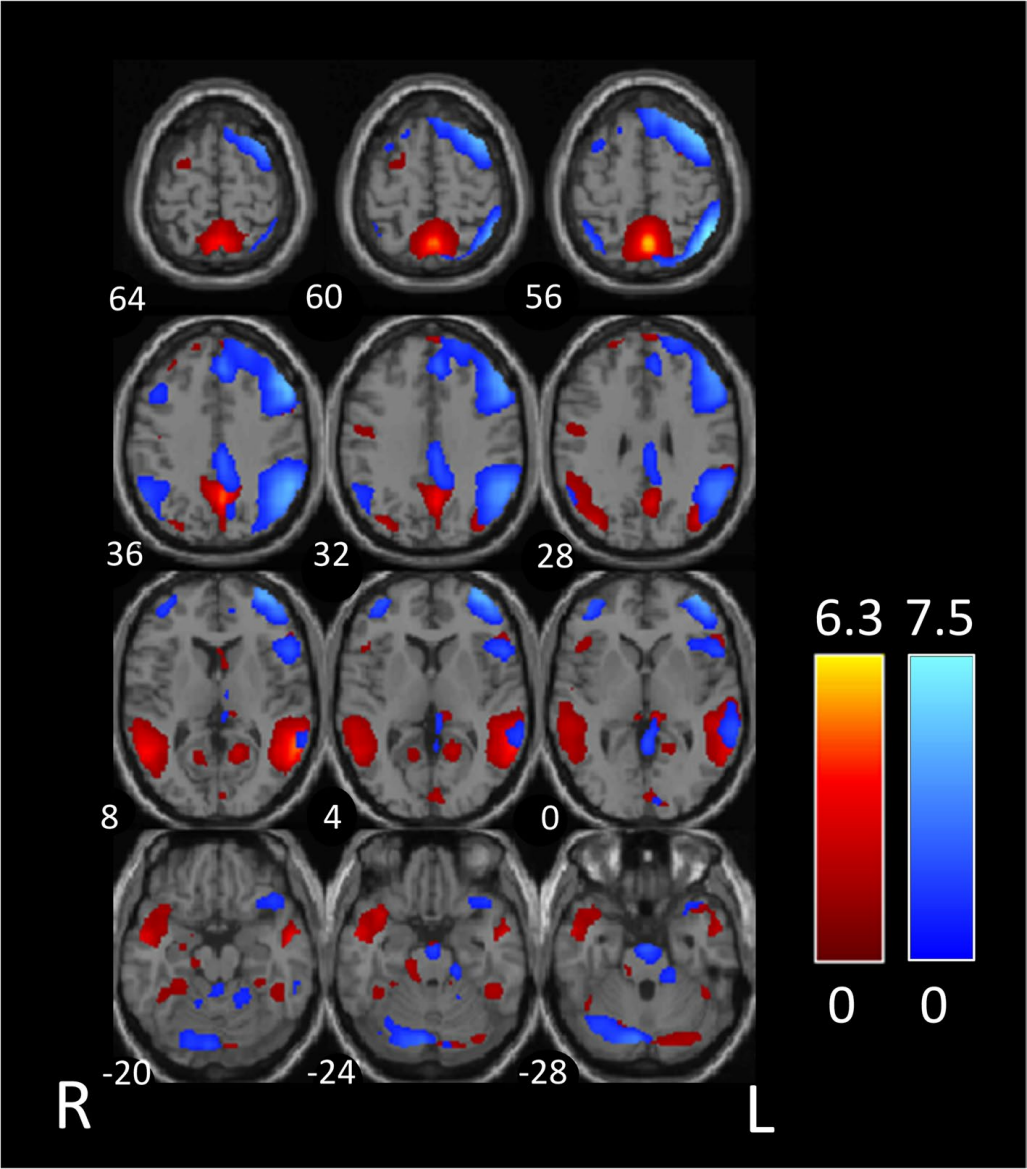
In the comparison between migraine with aura patients group and healthy subjects group before visual stimulation, the default-mode network (IC3, in red) and the left dorsal attention system (IC10, in blue) showed moderate connectivity. The images were co-registered in MNI space. The number under each brain image refers to the z-coordinate in Talairach space. The coloured bars reflect the functional connectivity maps (Z scores) of each network

The contrast between the HS group and the MwA group was slightly significant (*r* = 0.17, p = 0.018) at *p* < 0.05 FDR corrected (Fig. 1). No significant difference in lag was found between the 2 independent components.

In HS, we found that visual stimulation significantly increases functional connectivity between the independent components pair left DAS and ECN (*r* = -0.29; *p* < 0.001), and between right DAS and ECN (*r* = -0.29; *p* < 0.001) with no differences in lag.

In detail, before visual stimulation the correlations between left DAS and ECN (*r* = 0.17; *p* = 0.053), and between right DAS and ECN (*r* = 0.45; *p* = 0.692) were not significant.

After visual stimulation instead, left DAS and ECN (*r* = -0.02; *p* < 0.001), and right DAS and ECN (*r* = 0.27; *p* < 0.001) were significantly positively correlated.

**Fig. 2:**
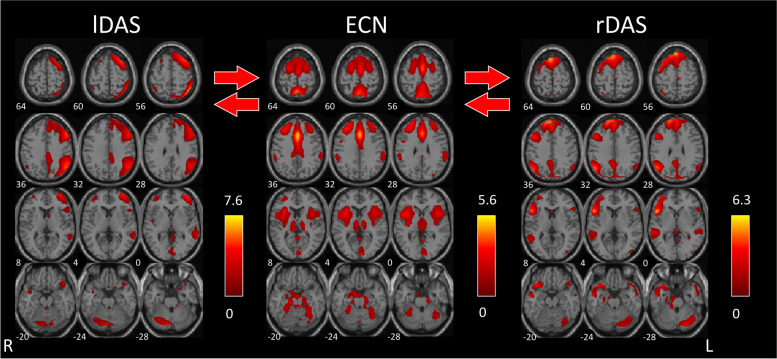
In healthy subjects, left [lDAS] and right [rDAS] dorsal attention systems were significantly connected to the executive control network [ECN], after 4-min checkerboard visual stimulation vs. baseline resting state network connectivity. The images were co-registered in MNI space. The number under each brain image refers to the z-coordinate in Talairach space. The coloured bar on the right side of each network component reflects its functional connectivity map (FDR-corrected at p < 0.01)

**Fig. 3:**
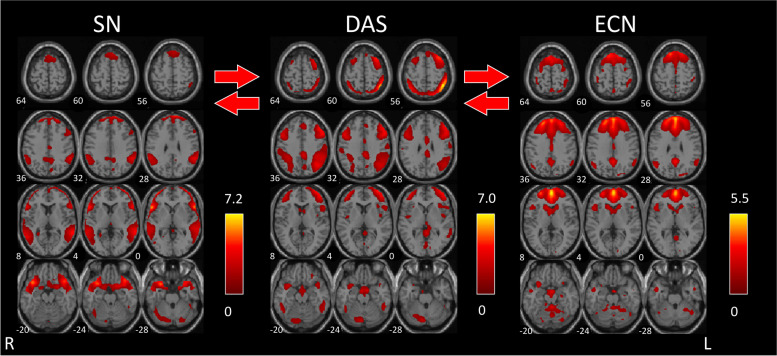
In patients with migraine with aura, dorsal attention systems [DAS] were significantly connected to the executive control network [ECN] and with the Salience network [SN], after 4-min checkerboard visual stimulation vs. baseline resting state network connectivity. The images were co-registered in MNI space. The number under each brain image refers to the z-coordinate in Talairach space. The coloured bar on the right side of each network component reflects its functional connectivity map (FDR-corrected at p < 0.01)

In patients with MwA, we found that visual stimulation significantly increased functional connectivity between the independent components pair SN and DAS (*r* = -0.39, *p* < 0.001, Fig. 2), and between DAS and ECN (*r* = -0.33, *p* < 0.001, Fig. 3) with no differences in lag. Before visual stimulation, the correlation between SN and DAS was not significant (*r* = 0.13; *p* = 0.016), whereas the positive correlation between DAS and ECN was significant (*r* = 0.18, *p* < 0.001). After visual stimulation, both pairs of components were significantly positively correlated (*r* = 0.51, *p* < 0.001; *r* = 0.51; *p* < 0.001).

### Relationship with clinical characteristics

In patients, correlation test reveals that after visual stimulation the Z-score of ECN correlated negatively with the monthly frequency of aura (*rho* = -0.544, *p* = 0.011). No other significant correlation was found between clinical and psychopathological variables, including mood and anxiety scale score, and resting networks strength.

## Discussion

In this study, we confirm in an independent group of MwA patients previous findings that functional connectivity between DMN and DAS is altered interictally compared to HS [16]. Here, instead of distinguishing patients on the basis of their aura, i.e. MwA with only visual aura or with associated somatosensory and dysphasic aura, we studied all patients in one group. We have previously pointed out that a lack of correlation between self-orientation monitoring networks, like DMN, and the externally-oriented multimodal sensory information processing networks, such as the DAS, may contribute to the emergence of pathology [16].

Our most striking finding however is that patients with MwA after 4-min visual stimulation present a different large-scale functional connectivity between cortical networks compared to healthy subjects. The results can be summarized as follows: comparing the recording made “after” vs “before” visual stimulation, healthy subjects show increased functional connectivity between the right and left DAS and the ECN, whereas MwA patients show increased functional connectivity of the DAS with both the SN and the ECN. The frequency of the aura was negatively correlated with the change in the strength of the intrinsic connectivity of the ECN collected after visual stimulation.

Human brain is organised in multiple large-scale neurocognitive networks that continuously interact with each other. Among other networks, the salience network has as key nodes the insula, antero-dorsal cingulate cortex, amygdala, and subcortical and limbic structures such as the thalamus and hypothalamus [26]. The SN is involved in many brain functions, the main one being the integration of sensory, emotional, and cognitive information [27]. In fact, the nervous system dynamically selects stimuli that have a specific valence among the enormous amount of incoming sensory input also thanks to the functionality of the SN. It receives converging inputs of different sensory nature, including visual ones [28]. The SN has both filtering and amplification functions for stimuli that might have an intrinsic biological salience, such as adaptation to repeated stimuli [27]. Another context in which the SN operates is that of selecting stimuli on which to focus attention and thus directing the resources necessary for goal-directed behaviour. These latter functions are carried out in concert with the functioning of other networks such as the DAS, anchored in the lateral frontoparietal cortex, and the ECN, anchored to the intraparietal sulcus and the frontal eye field [26]. Since our data show that immediately after the visual stimulation, the DAS in addition to the ECN, as observed in healthy subjects, is also connected with the SN, we believe that visual stimuli have a more emotionally and cognitively relevant meaning in MwA patients. The abnormal function of the SN in relation to other cortical networks such as the ECN has been repeatedly attributed to psychopathology [27]. However, our data do not show a direct correlation between anxiety and depression scales and the strength of resting-state networks.

Another possible explanation for the greater SN involvement in patients than in healthy subjects could be related to altered information processing at the thalamic level, being anchored to the SN and being a key hub between the brain and the peripheral inputs [28]. In line with this interpretation, several independent research groups have detected structural and functional abnormalities of thalamic sensory nuclei, their radiation and their connectivity with cortical networks, in MwA patients [4, 10, 13, 16, 29–35]. Previous studies show that patients with MwA may have an increased activation of the thalamus in visual responses [4], as well as high levels of photophobia, also in relation to the frequency of the aura [36]. Consequently, we cannot exclude that the present finding in MwA patients of an involvement of the SN, a centre of multisensory integration, may be due to the discomfort associated with viewing the visual stimulus. Nevertheless, only after the visual stimulation the correlation analysis shows that the higher the frequency of the aura, the lower strength of connectivity of the ECN. In previous studies, a lower activation of the ECN at rest may be associated with a lower capacity for planning and decision-making behaviour [27]. Consequently, we can only hypothesise that a higher frequency of migraine aura may lead to whilst normal-to-low functioning ability to access resources to behave towards cognitively relevant salient events such as aura-related transient focal neurological disorders. It remains to be investigated whether the previously observed recurrence of the aura-associated changes in cerebral blood flow not limited to the posterior vascular territories but spreading more anteriorly [37–39] can lead in the long run to functional changes in the ECN-anchored areas in MwA patients.

We acknowledge some limitations of this study. Firstly, we enrolled a relatively small group of subjects, which does not allow us to immediately generalise the results of the study. To assess reliability of our findings, future studies should be dedicated to trying to reproduce this initial evidence in an independent larger cohort of participants. Secondly, we did not include a group of patients with migraine without aura. This would have allowed us to ascertain whether SN involvement is specific to migraine with aura.

## Conclusions

To summarize, MwA patients differ from healthy subjects in cortical networking activity at rest after 4 min of visual checkerboard stimulation. Healthy subjects show increased connectivity between DAS and ECN, whereas patients show additional connectivity between DAS and SN. After visual stimulation, the strength of functional connectivity of the ECN depends on the frequency of the aura. To what extent these results are related to the recurrence of vascular insults from CSD or to abnormal processing of visual cues at SN and/or at the thalamic level, remains to be determined.

## Availability of data materials

The informed consent form signed by all participants in this study did not include a provision stating that individual raw data can be made publicly accessible. Therefore, in agreement with the Italian data protection law, individual de-identified participant raw data cannot be publicly shared. Researchers meeting the criteria for access to confidential data may access the data upon request.

## Abbreviations

BDI: Beck depression inventory
DAS: Dorsal attention system
ECN: Executive control network
FDR: False Discovery Rate
fMRI: Functional magnetic resonance imaging
HS: Healthy subjects
IC: Independent Component
ICA: Independent component analysis
MwA: Migraine with aura
RS: Resting state
SN: Salience network
STAI: State-trait anxiety inventory
TAS-20: Toronto alexithymia scale
